# A Fully Automatic Theorem Prover with Human-Style Output

**DOI:** 10.1007/s10817-016-9377-1

**Published:** 2016-06-11

**Authors:** M. Ganesalingam, W. T. Gowers

**Affiliations:** 1 Trinity College, Cambridge, UK; 2 Department of Pure Mathematics and Mathematical Statistics, University of Cambridge, Wilberforce Road, Cambridge, CB3 0WB UK

**Keywords:** ATP, Automated theorem proving, Human-oriented, Human-oriented theorem proving, Human-like output

## Abstract

This paper describes a program that solves elementary mathematical problems, mostly in metric space theory, and presents solutions that are hard to distinguish from solutions that might be written by human mathematicians.

## Introduction

### Overview of the Paper

The main purpose of this paper is to describe a program that solves elementary mathematical problems, mostly but not exclusively in metric space theory, and that presents the solutions in a form that is hard to distinguish from solutions that human mathematicians might write. The following two proofs are examples of the program’s output.^1^ The first is a proof that if $f:X\rightarrow Y$ is a continuous function and *U* is an open subset of *Y*, then $f^{-1}(U)$ is an open subset of *X*, and the second is a proof that if $f:X\rightarrow Y$ is an injection and *A* and *B* are subsets of *X*, then $f(A)\cap f(B)\subset f(A\cap B)$.

Let *x* be an element of $f^{-1}(U)$. Then $f(x)\in U$. Therefore, since *U* is open, there exists $\eta >0$ such that $u\in U$ whenever $d(f(x),u)<\eta$. We would like to find $\delta >0$ s.t. $y\in f^{-1}(U)$ whenever $d(x,y)<\delta$. But $y\in f^{-1}(U)$ if and only if $f(y)\in U$. We know that $f(y)\in U$ whenever $d(f(x),f(y))<\eta$. Since *f* is continuous, there exists $\theta >0$ such that $d(f(x),f(y))<\eta$ whenever $d(x,y)<\theta$. Therefore, setting $\delta =\theta$, we are done.

Let *x* be an element of $f(A)\cap f(B)$. Then $x\in f(A)$ and $x\in f(B)$. That is, there exists $y\in A$ such that $f(y)=x$ and there exists $z\in B$ such that $f(z)=x$. Since *f* is an injection, $f(y)=x$ and $f(z)=x$, we have that $y=z$. We would like to find $u\in A\cap B$ s.t. $f(u)=x$. But $u\in A\cap B$ if and only if $u\in A$ and $u\in B$. Therefore, setting $u=y$, we are done.

The main challenge we have faced in creating this program, discussed more extensively below, is that it does not seem to be possible to reconstruct genuinely human-like writeups from the proofs produced by automated provers from the machine-oriented tradition. In order to be able to produce such writeups we have had to use much more restricted proof methods than those available to modern provers. This in turn makes it more challenging for the prover to solve any individual problem, and indeed the program does not solve any problems that are beyond the reach of existing fully automatic provers. We should also note that we see this prover as the first stage of a long-term project to write a program that solves more complex problems in a ‘fully human’ way. We shall say a little about this wider project later in the paper.

For the remainder of this section, we shall discuss the human-style output further. We shall explain why we think it is a goal worth pursuing, and expand on the difficulties just mentioned. In Sect. 2 we shall discuss related work. In Sect. 3 we outline the main features of the prover. In Sect. 4 we describe the construction of the human-style writeup. In Sect. 5 we provide more extensive technical details about how the prover works, and in Sect. 6 we present a detailed example to illustrate how these technical elements operate in practice. In Sect. 7, we describe an informal experiment that we carried out in order to test whether the proofs produced by the program were indeed similar to the proofs that a human might write, and in Sect. 8 we provide some practical notes on running the program. Finally, in Sect. 9, we discuss possible future work.

### Why Bother with Human-Style Output?

These days there is a thriving subcommunity of mathematicians who use interactive theorem provers such as Coq, HOL, Isabelle and Mizar. However, it is also noticeable that the great majority of mathematicians do not use these systems and seem unconcerned about the possibility that their arguments are incorrect, even though there is plenty of evidence that incorrectness pervades the published literature.

There are several reasons for this, some of which are discussed by Bundy [14]. One is that the mathematical community has simply learnt to deal with the current state of affairs. Mistakes in the literature are less damaging than one might think, because either they are in papers that nobody wishes to build on in future work, or they are in papers that are important and tend therefore to be thoroughly scrutinized. Occasionally a paper by a reputable author makes an important claim that is very hard to verify because the paper is extremely complicated or badly written. In such situations, the paper will usually be treated with caution until some plausible confirmation of its correctness has been provided.

Another reason is that learning to use an interactive theorem prover, though not impossibly difficult, still requires an investment of time that most mathematicians are not prepared to make, when the reward is likely to be simply to tell them, after substantial effort, that a result is correct that they are already confident is correct. Of course, as just commented, sometimes mathematicians are *not* 100 % confident that a result is correct, and in such situations interactive theorem provers have an extremely valuable part to play: among their recent triumphs are verification of the four-colour theorem [18] and the Kepler conjecture [21]. Another remarkable achievement was the formalized proof of the odd order theorem of Feit and Thompson [19]: in that case the correctness of the theorem was not in serious doubt, but the paper of Feit and Thompson was very long and complicated and therefore difficult to check, so there is genuine value in having a formalized proof. This will be especially true if, as the authors hope, it leads to formalizations of other long and complicated proofs, of which there are an ever-increasing number. However, these notable examples are the exception rather than the rule, and the fact remains that most mathematicians do not feel that the benefits of automatic theorem provers are worth the effort when it comes to ‘normal’ mathematics.

So what *would* be beneficial to ‘typical’ mathematicians? One possible answer is to place far less emphasis on proofs as certifications of correctness and far more on proofs as *explanations*. Many mathematicians say that their aim, when looking for proofs, is to achieve understanding rather than to feel confident that a statement is true. Indeed, there are several sociological facts that cannot otherwise be explained. For example, why do mathematicians try to find proofs of results when a combination of experimental evidence and heuristic arguments establishes their truth beyond all reasonable doubt? (Goldbach’s conjecture is a statement that falls into this category). And why are new and different proofs of already known theorems sometimes highly valued? Why do mathematicians have a clear sense that some proofs are ‘better’ than others? It is clear that any answer to these questions, which have attracted considerable attention in the philosophical literature (see for example [26] and the references therein), has to take into account far more than the mere correctness of a proof.

Therefore, for an automatic theorem prover to be useful to mainstream mathematicians, it will be highly desirable for it to produce ‘good’ proofs that ‘explain what is going on’. Achieving this is of course a major challenge, but focusing on how humans find proofs is a good way of trying to meet that challenge, since there seems to be a close connection between the degree to which a proof is judged to be ‘explanatory’ and the ease with which one can see how somebody (a human, that is) might have thought of it. So an automatic theorem prover that imitates the way humans do mathematics is more likely to produce proofs that are appealing to human mathematicians.

For a proof to appeal to human mathematicians, a minimum requirement is that it should be written in a way that is similar to the way humans write. To design a system that produces human-style output one has a choice. One possibility is for the system to operate in a way that closely mirrors the way human mathematicians operate, in which case it is fairly straightforward to convert each step of its reasoning process into a piece of human-style prose, which will form the basis for the human-style output. The other possibility is for the system to operate in a quite different way from humans, but then to do some highly nontrivial processing to convert its initial logical output into a text that reads like what a human would write. The first of these options looks much easier, but it forces one to pay close attention to many details that would otherwise not matter. This gives rise to many interesting questions that can be regarded as easier cases of some of the fundamental questions in automatic theorem proving.

One such question is an obvious one that many people have asked: how do human mathematicians manage to avoid a combinatorial explosion when they search for solutions to complex problems? While we do not claim to have a satisfactory answer to this, we take the view (which is by no means universally shared, so this is a somewhat speculative claim) that a good way to look for the answer is to focus on exactly the kinds of small technical difficulties that have arisen in our work. In order to design a program that imitates human thought, we have had to place severe restrictions on how it operates; our hope is that if we can get these restrictions right, then we will have the beginnings of a program that can be scaled up to solve more complex problems with only a very modest amount of searching.

### Constraints and Challenges

As noted above, the main challenge we have faced is that we have not found it to be possible to ‘bolt on’ genuinely human-like output to an existing automated theorem prover. This seriously constrains the kind of methods that our prover can use. For example, a simple example of a procedure that is used by many theorem provers, especially those based on resolution, but that would be completely out of the question for us is putting all goals into conjunctive normal form. Not only do human mathematicians not do this, but the resulting search for a proof is quite unlike the kind of search that human mathematicians undertake, to the point where converting a successful search into a human-style write-up would be scarcely any easier than obtaining the write-up had been before the program started. The difficulty faced here is essentially the same difficulty found in decompilation of compiled code. In each case the high-level description (a human-style mathematical proof, a human-readable program) can always be converted into a low-level equivalent (a logical derivation, machine code). The translation is formally lossless, but at the same time some kind of higher-level structure that is important to humans is ‘lost in translation’ so that reversing the process, that is, converting machine code to a high-level program or a logical derivation to a human-style proof, is extremely difficult.

A less obvious example is the use of modus ponens. One might expect this to be fundamental to any theorem prover, but in fact pure modus ponens is almost never used by human mathematicians. Although generations of mathematicians are told that $P\implies Q$ means the same as $\lnot P\vee Q$, this does not in fact reflect the way mathematicians think or write (which causes genuine confusion to many first-year undergraduates). The implications that mathematicians actually use are almost always quantified ones— that is, implications of the form $\forall x\, (P(x)\implies Q(x)$) —although the quantification may not be explicitly mentioned.

Suppose, for example, that a human mathematician wishes to show that $3^{996}\equiv 1$ mod 997. A typical proof might read as follows: “Fermat’s little theorem states that if *p* is a prime and *a* is not a multiple of *p*, then $a^{p-1}\equiv 1$ mod *p*. Since 997 is prime, and 3 is not a multiple of 997, it follows that $3^{996}\equiv 1$.” A theorem prover that performed the deduction as follows would normally be regarded as acceptable:

1. $\forall a,p\in \mathbb {N}\ \ (p$ is prime  mod *p*).
2. 997 is prime.
.
4. 997 is prime  mod 997.
5. $$3^{997-1}\equiv 1$$ mod 997.
6. $$997-1=996$$.
7. $$3^{996}\equiv 1$$ mod 997.

In the above chain of reasoning, statements 1–3 are given as initial assumptions, 4 is obtained from 1 by universal instantiation, 5 is obtained from 2–4 by (unquantified) modus ponens, and 7 is obtained from 5 and 6 by equality substitution.

If we were to convert this reasoning directly into prose, it might end up something like this.

For every $a,p\in \mathbb {N}$, if *p* is prime and *a* is not a multiple of *p*, then $a^{p-1}\equiv 1$ mod *p*. Therefore, if 997 is prime and 3 is not a multiple of 997, then $3^{997-1}\equiv 1$ mod 997. But 997 is prime and 3 is not a multiple of 997. Therefore, $3^{997-1}\equiv 1$ mod 997. But $997-1=996$. Therefore, $3^{996}\equiv 1$ mod 997.

This is noticeably different from what a human would write, because of the second sentence, which corresponds to step 4. A human mathematician will deduce 5 from 1-3 using quantified modus ponens, so if we want to produce realistic human-style write-ups, we either have to emulate this or suppress the universal instantiation step when it comes to the write-up. But the latter option produces inappropriate proofs in other cases: for example, if steps occur between the universal instantiation and modus ponens, then the writeup can end up implicitly using a fact which has not been mentioned for some time, which contravenes rules regarding the coherence of discourse and so generates an unnatural writeup.

Another striking and important feature of human mathematical reasoning, which is reflected in some but not all theorem provers, is that it tends to take place at as high a level as possible, at least when it is done well. For example, an inexperienced undergraduate, when asked to prove that the graph of the function $f(x)=x^2$ is a closed subset of $\mathbb {R}^2$, might well choose a point (*x*, *y*) in the complement of the graph and laboriously find $\epsilon >0$ such that no point (*z*, *w*) within $\epsilon$ of (*x*, *y*) belonged to the graph. A more experienced mathematician would give a high-level proof such as this.

Let $g(x,y)=x^2-y$ for each $x,y\in \mathbb {R}$. Then *g* is a continuous function and the graph of *f* is the inverse image $g^{-1}(\{0\})$. Since the singleton $\{0\}$ is closed, so is $g^{-1}(\{0\})$.

This short argument illustrates several of the kinds of phenomena that concern us. The main one we wish to highlight is that it does not make any use of the definition of ‘is closed’ beyond the fact that in order to apply a general result about inverse images of closed sets one needs a match for the hypothesis ‘*A* is closed’. In particular, at no point does the argument perform a definition expansion of the statement ‘$\{0\}$ is closed’.

Another feature of the argument is that although it is logically necessary to justify the statement that *g* is continuous, it will be acceptable to many mathematicians not to do so, since the continuity of *g* is ‘more basic’ than the statement being proved. (Similarly, if one *were* writing out a proof of this easier result, one would normally rely on yet more basic statements such as the continuity of $f(x)=x^2$ and of the coordinate projections from $\mathbb {R}^2$ to $\mathbb {R}$). A similar remark applies to the statement that the graph of *f* is equal to $g^{-1}(\{0\})$ and the statement that $\{0\}$ is closed.

A different phenomenon is illustrated by the last sentence: it uses the result that the inverse image of a closed set under a continuous function is closed without explicitly mentioning it. It really does feel to a mathematician as though if a function *g* is continuous and a subset *A* of its range is closed, then those two facts together imply that $g^{-1}(A)$ is closed. This implication is ‘mathematical’ rather than ‘purely logical’. Of course, one can analyse it logically by explicitly stating the theoretical result being used and applying quantified modus ponens, but that is a temptation one needs to resist if the program is to produce human-style write-ups without an undue amount of processing.

There are several phenomena like these, and our main challenge has been to identify and take account of them when writing our program. A subsidiary challenge has been ordering the different tactics that humans use in order of attractiveness; if this is not done correctly then the prover will still find proofs but those proofs will read as ‘odd’, because they follow a line that seems strange to a human.

There is one further constraint that needs some justification, which is that our program does not have any ability to backtrack: if it tries out a sequence of steps and gets stuck, then it simply stops. This might seem to be a flaw with the program, since human mathematicians definitely *do* backtrack. However, we took the decision to concentrate on routine problems, which we define informally as precisely those for which humans do not consciously backtrack, since it seems important to have a program that performs well on routine problems before one tackles the formidable question of just how humans limit their searches when the problems become more difficult. Fortunately, there are many proofs that an experienced mathematician will remember as ‘Just do the obvious thing at each stage and it all works out.’ Our initial aim (which we are still a long way from achieving) was to write a program that would solve all routine problems in the way that humans would solve them, which in particular means without backtracking.

## Related Work

### Systems with Natural-Language Output

Several systems have been developed that use natural language to a greater or lesser extent. An early example with some similarity to ours is that of Felty and Miller [16]. They start with a proof tree and convert it into a more readable form. Their system can also make significant changes to how a proof is presented. The following is an example of output from their system: it is a proof that there are infinitely many primes. The function *f* mentioned in the proof can be taken to be the function defined by the formula $f(n)=n!+1$: then the beginning of the proof is asserting some properties of this function that are subsequently used in the proof (so any other function with those properties would do just as well).

Assume $\forall x (f(x)>x)\wedge \forall x\forall y(\mathrm {div}(x,f(y))\supset (x>y))\wedge \forall x(\lnot \mathrm {prime}(x)\supset \exists y(\mathrm {prime}(y)\wedge \mathrm {div}(y,x))$. We have two cases. Case 1: Assume $\lnot \mathrm {prime}(f(a))$. By modus ponens, we have $\exists y(\mathrm {prime}(y)\wedge \mathrm {div}(y,f(a)))$. Choose *b* such that $\mathrm {prime}(b)\wedge \mathrm {div}(b,f(a))$. By modus ponens, we have $(b>a)$. Hence, $(b>a)\wedge \mathrm {prime}(b)$. Thus, $\exists x((x>a)\wedge \mathrm {prime}(x))$. Case 2: Assume $\mathrm {prime}(f(a))$. Hence, $(f(a)>a)\wedge \mathrm {prime}(f(a))$. Thus, $\exists x((x>a)\wedge \mathrm {prime}(x))$. Thus, in either case, we have $\exists x((x>a)\wedge \mathrm {prime}(x))$. Since *a* was arbitrary, we have $\forall n(\exists x((x>n)\wedge \mathrm {prime}(x)))$.

They describe their mechanism for converting the original tree-structured deductions into readable natural-language text as very simple. It is clear that with some small changes they could have improved the readability. For example, they could have replaced $\mathrm {prime}(x)$ by ‘*x* is prime’, $\mathrm {div}(x,y)$ by *x*|*y* and the symbols for connectives by English words. However, the result would still have had some slightly odd characteristics—for instance, no human mathematician would bother to write ‘by modus ponens’—that would have betrayed its mechanical origins.

Another program that produced readable text was written by Holland-Minkley et al. [22]. Their aim was to create natural-language output from the Nuprl system. This is an interactive system based on tactics that is designed to mimic human reasoning. The output from the Nuprl system is not at all easy for the untrained mathematician to read. However, they could convert it into language that was considerably closer to what a human mathematician might write, as the following sample demonstrates. (We have slightly modified what they wrote, replacing pairs of minus signs by the cutoff subtraction symbol $\dot{-}$, which seems to be what was intended).

Theorem: For integers *a* and *b* and natural number *c*, $(a\ \dot{-}\ b)\ \dot{-}\ c = a\ \dot{-}\ (b + c)$. Consider that *a* and *b* are integers and *c* is a natural number. Now, the original expression can be transformed to $\mathrm {imax}(\mathrm {imax}(a - b; 0) - c; 0) = \mathrm {imax}(a - (b + c); 0)$. From the add com lemma, we conclude $\mathrm {imax}( -c+\mathrm {imax}(a + -b; 0); 0) =\mathrm {imax}(a + -b + -c; 0)$. From the imax assoc lemma, the goal becomes $\mathrm {imax}(\mathrm {imax}((a + -b) + -c; 0 + -c); 0) = \mathrm {imax}(a + -b + -c; 0)$. There are 2 possible cases. The case $0 + -c \le 0$ is trivial. Consider $0 < 0 + -c$. Now, the original expression can be transformed to $\mathrm {imax}((a + -b) + -c; 0 + -c) = \mathrm {imax}(a + -b + -c; 0)$. Equivalently, the original expression can be rewritten as $\mathrm {imax}((a + -b) +-c) = \mathrm {imax}(a +-b +-c; 0)$. This proves the theorem.

In places this looks like the kind of continuous prose that a mathematician would write, though as with Felty and Miller’s system there are a number of telltale signs of the mechanical origins of the text. For instance, the first sentence is not quite grammatical: a human would write, ‘Let *a* and *b* be integers and let *c* be a natural number.’ There is also the trivial point that mathematicians would write ‘max’ rather than ‘imax’ (trivial because it would be very easy to change this). There is also a repetitive quality to the prose that gives it an automatically generated feel: for instance, two sentences open with ‘Now, the original expression can be transformed to’.

There are further differences that highlight a point that will be important to us later. For example, when proving that $A=B$ via a string of intermediate inequalities, mathematicians will normally express the proof in the form $A=A_1=\dots =A_k=B$. From the write-up above, it is clear that Nuprl prefers to deal with equivalences between statements: a typical step might be to reduce the original goal $A=B$ to the goal $A=A_k$, for instance.

Another difference is that the proof makes use of terms that a human would consider too ugly and unnatural to write. For example, no human mathematician would ever write “The case $0+-c\le 0$,” instead writing the condition in the more obvious way as “$c>0$”.

It is perhaps helpful to distinguish between two kinds of unnaturalness of a program’s output: roughly speaking, unnaturalness in how the program expresses its thoughts, and unnaturalness of the thoughts themselves. Writing ‘imax’ and $a+-b$ are examples of the former, while avoiding equational reasoning and considering expressions with strange terms in them are examples of the latter.

A further example of the latter, which we touched on in the previous section, is use of tiny lemmas. Presumably ‘the imax assoc lemma’ is the statement that the binary operation of taking the maximum of two numbers is associative. This statement belongs to a troublesome class of statements that human mathematicians will normally never state explicitly, even when they first meet the basic definitions. The precise reason for this is unclear, but it appears to be something like that we visualize the maximum of a finite set of numbers as its rightmost element on the number line and can immediately see that that rightmost element will win a knockout competition however the competition is organized.

Another example of a statement in this class is the general principle that if a binary operation $\circ$ is associative, then the expression $x_1\circ \dots \circ x_k$ is unambiguous. (More precisely, however you bracket it, you will get the same result). The associativity assumption itself is just the case $k=3$, and yet generations of mathematicians happily use the more general principle that brackets are unnecessary without ever stopping to prove it.

A third system that has aims that are in certain respects similar to ours is the Theorema system of Buchberger et al. [13]. However, we ask significantly more of our output than the developers of Theorema: for them it is sufficient that the output should be comprehensible to mathematicians, whereas we ask that it should be hard to distinguish from what a human might write. For example, this is the statement of a lemma in the Theorema language (quoted in the paper referred to).

#### Lemma

(“coprime”, any[*a*, *b*] with[nat$[a]\wedge$nat[*b*]], $2b^2=a^2\implies \lnot$coprime[*a*, *b*])

A human rendition of this lemma would read more like this.

#### Lemma

Let *a* and *b* be natural numbers satisfying the equation $2b^2=a^2$. Then *a* and *b* are not coprime.

In other words, it would be written in continuous prose, albeit prose of a characteristic ‘semi-formal’ kind, which makes it significantly easier to read.^2^

This leads us to an important general point about all three of the systems discussed. The output from these systems is comprehensible to mathematicians, in the sense that they can decipher it. But it is hard work, enough so to prevent most mathematicians from using these systems. (It seems likely that one becomes used to reading proofs like the ones above with exposure. But we suspect that this same factor leads those who use theorem provers regularly to underestimate the uphill struggle a typical mathematician faces when reading the proofs like the ones above).

Part of the difficulty in reading these proofs is due to an overreliance on symbols; in proofs written by humans, symbolic material plays a very specific and limited role [17], which does not include many of the uses in the example above. But a larger part relates to higher-level structure: none of the proofs presented above *flow* like real mathematical proofs. Mathematical proofs are like natural language texts—such as the document you are reading—in that sentences link together to form coherent discourses. From a mathematician’s perspective, this is what allows a proof to read as presenting a coherent argument rather than merely a sequence of facts. The principles behind discourse structure have been extensively studied by linguists (see e.g. [1]). We do not know of a way of tacking on this kind of structure, which is key to readability, to the proofs presented in this section; in order to produce it we have had to design our prover to support it from the ground up. In particular, the prover operates in a way that models a human closely enough to let it suppress irrelevant information and produce much larger blocks of discourse-coherent text than in the proofs given above.

### Other Related Work

The constraints involved in producing human-like writeups mean that our prover functions in a very similar way to some older human-oriented provers produced by Woody Bledsoe and his students [2, 5–11]. His *Non-resolution theorem proving* [6] outlines the main concepts involved in human-oriented theorem proving; a number of these are very relevant to the work we describe below, including the use of rewrite rules, forward chaining, (a kind of) typing, a reluctance to expand definitions unless necessary, and the use of ‘natural systems’. Further, as we shall describe below, our system shares the ‘waterfall architecture’ used in the provers created by Bledsoe’s students Boyer and Moore [12].

aAlthough human-oriented proving has largely fallen out of fashion, there have been some more recent systems that mimic natural proof styles. The most notable of these is Weierstrass [3], which is capable of generating $\epsilon$-$\delta$ proofs. One distinctive feature of this system is as follows:

The intention [of Weierstrass] is, to produce a proof that can be read and checked for correctness by a human mathematician; the standard to be met is “peer review”, just as for journal publication. By contrast, the purpose of Weierstrass is *not* to produce formal proofs in a specified formal system.

As we shall discuss more extensively in Sect. 3, the prover we are describing here has exactly the same goals as Weierstrass: it aims to produce proofs for human consumption, not proofs that are formally certified correct.

The article just cited does not provide any actual $\epsilon$-$\delta$ proofs, noting simply that “the strict length limit does not permit the inclusion of the actual output of Weierstrass”. Similarly an article on the irrationality of *e* [4] does not contain any actual output. We have also been unable to obtain the program and are therefore not able to comment on how human-like the output of Weierstrass actually is.

Another recent human-oriented system is Analytica [15], a theorem prover built inside Mathematica. The output of this system contains some words, but it is much less human-like than even the systems described in the previous section; essentially it prints out a long sequence of equations connected by phrases such as ‘reduces to’ and ‘rewrites as’. The result is not written in sentences, let alone being grammatical, so we do not classify Analytica with the systems described in the previous section. (This is not a criticism of Analytica, as its authors make no claim to produce human-readable output).

We should emphasize that the work we describe is not meant to be competitive with any of the provers described here when considered as a prover; as noted above, the additional constraints involved in generating human-readable writeup are important to our project, but they also rule out many sound and effective tactics. Thus, our system is operating at a disadvantage, though it is our hope that ultimately the extra work we have to do now will help us see how to design systems that are more powerful than existing systems at solving the kinds of problems that human mathematicians are good at.

There is also an extensive literature on systems that accept natural language-like *input*, including MIZAR [31], NaProChe [25], ForTheL [32] and MathNat [23]; we will not discuss these further here because the acceptance of human-like input and generation of human-like output are, from a linguistic perspective, almost unrelated problems. Additionally most systems of this kind focus on checking that proofs are correct, which is (as discussed above) not a concern for most human mathematicians. Thus, they serve a different purpose from that of the work we are describing here.

## Key Features of the Prover

The basic architecture of our prover is, as we have mentioned, very similar to the ‘waterfall’ architecture used in the Boyer-Moore provers. The program contains a number of heuristics that transform the goal in a sound manner. These heuristics are ranked by attractiveness. The program operates fully automatically by repeatedly applying the most attractive heuristic that has an effect. The underlying logic is essentially first-order logic with equality, though there are some caveats to be made here (in particular involving structural sharing of formulae and the use of metavariables) which we will outline as we go through the paper.

Notwithstanding the overall waterfall architecture, the details of the operation of the program are actually better described with reference to the goal-oriented tactic-based style of proof used in some interactive LCF-style theorem provers [28]. The correspondence may be drawn simply by regarding each heuristic as a tactic; the program can then be thought of as repeatedly applying a single tactic, which is itself constructed by taking a list of subsidiary tactics and applying the first that can be applied. In the remainder of the paper we will refer to tactics rather than heuristics, not least because we do not expect the waterfall architecture to be the last word on selecting tactics for a system of this kind.

We should stress that there is a key respect in which our system is not LCF-like. Like the Weierstrass system described in Sect. 2, it is not attempting to certify proofs correct in the way that LCF provers do; there is no small ‘safe’ kernel. Although we have every respect for the goal of certification, our concern in this paper is the generation of human-like proofs.

While it would certainly be possible to produce a program that generated human-like proofs and certified them correct, this is not an easy exercise for a number of reasons. One of these relates to the different number systems in use in mathematics: most work in theorem proving treats, say, the natural number 3 as being distinct from the rational number 3/1, whereas human mathematicians consistently treat them as the same object. One way of seeing this is to consider a standard definition like

$$\begin{aligned} \ ^nC_r = \frac{n!}{r!(n-r)!} \end{aligned}$$

in which the definiendum is a natural number but the definiens is a rational number. Handling the number systems in a human-like fashion in a theorem prover is far from straightforward because under standard accounts, the natural numbers are *used* to define the integers and thence the rational numbers. A full treatment requires an analysis of the operation of identification, which in turns requires modifications to the underlying logic [17].

In the remainder of this section, we highlight some other significant respects in which our approach differs from more typical approaches.

### Targets, and the Structural Sharing of Goals

Rather than having a list or stack of goals, we have a single goal, which may be regarded as a structurally shared version of the collection of goals found in a prover such as HOL. This structural sharing is necessary both for efficiency and in order to model human reasoning processes closely enough to produce human-like write-ups. A human reasoner will generally keep track of a list of background ‘assumptions’ which are known to be true (and likely to be helpful) and a separate list of statements that need to be proved. Under many circumstances the human will reason forwards from the assumptions without knowing in advance which of the statements to be proved will be deduced. If each goal were represented independently, this kind of forwards reasoning would have to be performed more than once. Thus, in our system the goal consists of a list of assumptions and a list of statements to be deduced from those assumptions, which we refer to as *targets*. If we represent the goal as a sequent $A_1,\dots ,A_m\vdash B$, where *B* is of the form $B_1\wedge \dots \wedge B_k$, then the targets are the statements $B_i$.

We should emphasise that our targets are not the same as the consequents $C_1,\dots ,C_k$ of a sequent $A_1,\dots ,A_m\vdash C_1,\dots ,C_k$; consequents are interpreted disjunctively, whereas targets are to be interpreted conjunctively. Thus our sequents always have a single consequent, and the conjuncts of that consequent are the targets. This is a good illustration of our priorities. Consequents may be more attractive from a logical point of view for symmetry reasons, but the convention we adopt, where we list the assumptions and targets, is in our judgment closer to how humans would think of ‘the current state of play’ when they are in the middle of solving a problem, and that is more important to us than logical neatness.

### The Library

Like many other systems, the program also has a library of statements that it may assume. However, the role that our library plays is very different. With an interactive program whose main aim is proof verification, the library will typically be a huge database of statements that have already been fully checked and can therefore be used safely.

By contrast, for us the library is provided by the user and represents a body of results and definitions that a human mathematician would know and feel free either to quote or to use silently when trying to solve the problem at hand. Thus, the question of whether or not a statement is appropriate for our library is one that has to be considered carefully, and the answer varies from problem to problem. For example, if the system is asked to prove the associativity of set intersection, then we do not want it to say, ‘This result is already in the library, so we are done.’ But for a more advanced problem, we want the associativity of set intersection to be available to the program (or perhaps even built in to its reasoning processes) rather than requiring the program to formulate and prove it as a lemma. As ever, this is because we are trying to imitate human mathematical thought and so generate human-like output: one of the things an undergraduate mathematician has to learn is what it is appropriate to assume when proving a result.

Thus, to use the program, it is not usually enough simply to input the goal and hope that the program will manage to prove it. Typically, the user has to add appropriate background results to the library first.

The library that we have used with the problems we have worked on is small, so we have not had to face the potential problem of the program using a result that is ‘more advanced’ than what it is supposed to be proving. However, when this problem does arise, as it inevitably will, we plan to order our library results using a relation ‘is more advanced than’, so that for each problem we can simply instruct the library not to use results beyond a certain point in the ordering.

We have also adopted a policy of being sparing about what we allow in the library. Human mathematicians do not store all the true mathematical statements that they come across in their brains. Rather, they seek out statements or techniques that have a kind of general quality. Exactly what this quality is, we do not claim to understand. It may be that for a more sophisticated program one should not expect to be able to judge the appropriateness of a statement in advance, but should store promising statements and then gradually forget about them if they turn out not to be especially useful.

Two examples of results that we have in the library we currently use are the statement that a closed subset of a metric space contains its limit points, and transitivity of ${<}$. Actually, it is convenient to store four separate transitivity statements such asone for each way of choosing ${<}$ and $\le$. This saves the program from rederiving these variants from the transitivity of ${<}$. Once again, this is because we are modelling human thought: a typical human mathematician will internalize all four transitivity statements and use them without thinking. It would look very strange in a write-up if the program stopped to prove the above transitivity statement as a lemma.

A statement such as the triangle inequality would be most usefully stored in the library in the following form.In this form it is appropriate for most simple deductions, and reflects quite well how human mathematicians use the inequality. However, we do not yet have a satisfactory understanding of the process whereby human mathematicians are told the triangle inequality in the usual form

$$\begin{aligned} d(x,z)\le d(x,y) + d(y,z) \end{aligned}$$

and then quickly use it to have thoughts such as, ‘I need *d*(*x*, *z*) to be less than $\gamma$, so it will be enough to ensure that $d(x,y)<\gamma /2$ and $d(y,z)<\gamma /2$.’ That is not to say that we cannot think of a mechanical method that would manage to make a deduction like this: the difficulty comes when we try to understand (in order to imitate) how humans do it.

At the moment, the library contains four kinds of data:

1. Results, i.e. facts that the program can utilise while constructing a proof.
2. Definitional expansions.
3. Term rewrite rules.
4. Constructions.

Results and (definitional) expansions are the key to reasoning at a high level, which is in turn one of the keys to producing human-like proofs. Term rewrite rules and instructions play a much smaller role in the program. The former are used to change e.g. $(f\circ g)(x)$ into *f*(*g*(*x*)), which is an instinctive and automatic step for any human mathematician. Constructions generally ‘finish off’ a problem by supplying an object with certain properties that are being looked for. For example, suppose the program needs to find *x* s.t. $x \leqslant a$ and $x \leqslant b$; the library can be used to construct $\min \{a, b\}$, which has the necessary properties.

### Removal of Assumptions

Another unusual aspect of our approach involves paying close attention to the removal or deletion of assumptions. We include tactics for removing assumptions not because they have a significant effect on the output of the program, but because of our commitment to modelling how human mathematicians think. The particular phenomenon we are trying to model is that when humans work out proofs, they often find it obvious that a statement has been ‘used up’ and will have no further role to play in the proof. We have modelled this by incorporating tactics that, under appropriate circumstances, remove assumptions from the list of all assumptions.

One reason we are interested in the removal of assumptions is that it forces us to think about a relatively simple special case of a hard and important general problem in theorem proving, namely the problem of deciding which statements are likely to be relevant to the solution of a given problem. It is possible to write programs that search through very large numbers of statements until they find something that magically works, but humans do not do this. So we feel that any efforts we make to understand this aspect of how humans do mathematics will pay dividends when we try to scale up to systems that will find more complex proofs. Another advantage of including tactics that remove assumptions is that it makes it considerably easier to debug cases where the program is stuck, by removing a lot of the ‘noise’ that makes it hard to understand intermediate goals.

## Writing Up

In general, natural language generation is a complex process. It involves multiple levels of planning, which draw on both domain knowledge and models of the intended audience, and also a phase when the actual text is generated, which draws on syntactic, morphological and lexical information. An overview of the process may be found in [29]. Because of this complexity, building a fully fledged natural language generation system is a major task. Furthermore, since mathematics contains not just English words but also a large array of distinctive symbols used in distinctive ways, it is not at all straightforward to use off-the-shelf systems.

Fortunately, mathematical language has properties that make the task considerably simpler than it is for the English language in general. Foremost among these is the fact that mathematical proofs almost always have a particularly simple rhetorical structure. To some degree this is because the domain of discourse includes only timeless facts, which itself rules out a large proportion of the rhetorical relations found in general text. But the main reason is that there is a strong convention that further constrains the rhetorical structure of proofs. A proof proceeds by the presentation of a sequence of assertions, each of which follows from the premises of the theorem being proved or from previous assertions. This structure is not accidental; it is a direct reflection of the fact that mathematicians process proofs by reading and verifying one sentence at a time, and would not expect the justification of a fact presented in one sentence to be deferred to a later sentence. (We are talking here about proofs of the level of simplicity of the proofs discussed in this paper. For more complicated arguments, facts may sometimes be used before they have been proved, but in good mathematical writing this will be carefully flagged up to make it as easy as possible for the reader to check that the resulting argument is complete and not circular).

This convention gives us an easy way to produce write-ups of our proofs. An obvious strategy is to allow each application of a tactic to generate some number of sentences (possibly zero), and then to concatenate the output from the different tactics to produce the final text. Note that this strategy is viable only because we are absolutely rigorous about requiring our tactics to reflect steps in human reasoning; in effect, the strategy is mimicking a human who is carefully writing down a proof while coming up with it, which is quite straightforward for an experienced mathematician. (Again, this becomes less true if the proofs are more difficult). As we shall see below, this simple strategy produces surprisingly good results, though with a weakness that needs to be dealt with by a postprocessing phase that turns out to be straightforward.

Because we have a fixed list of tactics, implementing the strategy only requires us to specify which sentences (if any) are produced for the applications of each tactic. A very simple way to do this is to use *template generation*: each tactic is associated with a *template*, or ‘piece of text with holes’, and the holes are filled in with concrete information about the facts and objects used in the actual application. So, for example, forwards reasoning may be associated with a very simple template ‘since ${<}{} \textit{facts}{>}$, ${<}deduced~fact{>}$’. Instantiating this template would produce text like

Since *A* is open and $x\in A$, there exists $\eta > 0$ such that $u\in A$ whenever $\textit{d}(x,u) < \eta$.

Note that individual facts are expressed in idiomatic ways, rather being displayed in a way that directly reflects the underlying predicate calculus; thus we have ‘*A* is open’ and ‘$\eta > 0$’ rather than ‘$\textit{open}(A)$’ and ‘${greater\_than}(\eta , 0)$’. The same is true of objects: we display ‘$f \circ g$’ rather than $\textit{compose(f,g)}$, and so on. Similarly quantification is expressed idiomatically using words like ‘whenever’, where possible, rather than using more stilted phrases like ‘for all’, which would more directly reflect the underlying predicate calculus.

An example of the text produced by this method is as follows:

Let *x* be an element of $A\cap B$. Since $x\in A\cap B$, $x\in A$ and $x\in B$. Since *A* is open and $x\in A$, there exists $\eta >0$ such that $u\in A$ whenever $d(x,u)<\eta$. Since *B* is open and $x\in B$, there exists $\theta >0$ such that $v\in B$ whenever $d(x,v)<\theta$. We would like to find $\delta >0$ s. t. $y\in A\cap B$ whenever $d(x,y)<\delta$. But $y\in A\cap B$ if and only if $y\in A$ and $y\in B$. We know that $y\in A$ whenever $d(x,y)<\eta$. We know that $y\in B$ whenever $d(x,y)<\theta$. Assume now that $d(x,y)<\delta$. Since $d(x,y)<\delta$, $d(x,y)<\eta$ if $\delta \le \eta$. Since $d(x,y)<\delta$, $d(x,y)<\theta$ if $\delta \le \theta$. We may therefore take $\delta =\min \{\eta ,\theta \}$. We are done.

The main problem with this text is that it suffers a lack of coherence, in the sense defined in [24]: the sentences are individually acceptable, but they do not combine to form an idiomatic discourse. The principal reason for this is that the text repeats information unnecessarily. For example, in

Since $x\in A\cap B$, $\underline{x\in A}$ and $x\in B$. Since *A* is open and $\underline{x\in A}$, there exists $\eta > 0$ such that $u\in A$ whenever $\textit{d}(x,u) < \eta$.

the repetition of the underlined phrase is awkward. Because it is introduced by the sentence immediately preceding the ‘since’ clause, it is awkward to have it spelt out explicitly within that clause. Similarly, consider:

Since $\underline{\textit{d}(x,y) < \delta }$, $\textit{d}(x,y) < \eta$ if $\delta \le \eta$. Since $\underline{\textit{d}(x,y) < \delta }$, $\textit{d}(x,y) < \theta$ if $\delta \le \theta$.

Here, having two identical ‘since’ clauses in consecutive sentences is again awkward: the repetition of material is unwieldy and unidiomatic.

We are of the opinion that [24] correctly diagnoses the underlying problem here: spelling out rhetorical relations, or aspects of rhetorical relations, that can easily be inferred from the context violates Grice’s maxim of quantity [20]. Often the solution is to substitute an appropriate and less explicit *cue phrase*. For example, ‘since *A* is open and $\underline{x\in A}$, ...’ is better replaced by ‘therefore, since *A* is open, ...’. The cue phrase ‘therefore’ (which assumes that the relevant reason has just been given) is less explicit than the cue phrase ‘since’ (which subordinates an explicitly stated reason), so it avoids spelling out information that is clear from the context. In other cases repetition can be avoided by combining sentences; thus the previous example may be changed into

Since $\underline{\textit{d}(x,y) < \delta }$, $\textit{d}(x,y) < \eta$ if $\delta \le \eta$ and $\textit{d}(x,y) < \theta$ if $\delta \le \theta$.

The initial ‘sentence by sentence’ process described above is followed by a series of transformations that manipulate pairs of consecutive sentences in order to resolve the issues just mentioned. (Needless to say, the transformations operate on a structural level rather than on the literal text). Applying this series of transformations to the example text above yields:

Let *x* be an element of $A\cap B$. Then $x\in A$ and $x\in B$. Therefore, since *A* is open, there exists $\eta >0$ such that $u\in A$ whenever $d(x,u)<\eta$ and since *B* is open, there exists $\theta >0$ such that $v\in B$ whenever $d(x,v)<\theta$. We would like to find $\delta >0$ s. t. $y\in A\cap B$ whenever $d(x,y)<\delta$. But $y\in A\cap B$ if and only if $y\in A$ and $y\in B$. We know that $y\in A$ whenever $d(x,y)<\eta$ and that $y\in B$ whenever $d(x,y)<\theta$. Assume now that $d(x,y)<\delta$. Then $d(x,y)<\eta$ if $\delta \le \eta$ and $d(x,y)<\theta$ if $\delta \le \theta$. We may therefore take $\delta =\min \{\eta ,\theta \}$ and we are done.

One particular point worth emphasising is that the write-up process is deterministic: it will always produce the same output text for any given proof. This is for two reasons. First, if any non-determinism had been present we would have had to evaluate many outputs for any given proof, which would have made iterative improvement and fine-tuning of the write-ups considerably slower. Secondly, and more importantly, if the process were nondeterministic, our claim that the program produced human-like output would be suspect, in that we would have been able to run the program several times and ‘cherry pick’ output. Unfortunately, this determinism has an undesirable (but fully anticipated) side-effect. When one compare several proofs produced by the program, the write-ups are much more similar than those a human would produce. For example, most proofs produced by the program end with the phrase ‘we are done’. In the long run, we will undoubtedly need to introduce nondeterministic stylistic variation, allowing the program to vary the text generated for a particular step in just the way human would, despite the difficulties that will cause.

Finally, it is worth noting that during the evaluation process described in Sect. 7, we collated a wealth of data on how humans write up proofs. We anticipate using this data in combination with carefully chosen natural language processing techniques to create substantially improved versions of the write-up procedure.

## Technical Details

### Formalism

The formulae used in the program are essentially formulae of first-order logic, augmented with metavariables. More formally, we have:

1. A collection of *functions*, each with a distinct name and an (implicit) arity. An example is *compose*.
2. A collection of *predicates*, each with a distinct name and an (implicit) arity. An example is *less_than*. Equality is represented by a distinguished predicate.
3. A collection of *mathematical types*, each with a distinct name. An example is *positive real number*. At the moment the collection of types is specialized for use in problems involving metric spaces.
4. A *variable* is specified by a name (typically a single character), a mathematical type and a ‘variable type’, which indicates whether or not the variable is a normal variable or one of two kinds of metavariable, discussed in Sect. 5.8 below. Variables are also annotated with information indicating that certain variables are independent of other variables, which constrains inference.
5. A *term* is either a variable or a function together with a list of terms of appropriate arity.
6. An *atomic formula* consists of a predicate together with a list of terms of appropriate arity.
7. A *formula* consists of one of the following, where $v_i$ are variables and $F_i$ formulae:
  An atomic formula.
$$\lnot F_1$$
$$F_1 \vee F_2$$
$$F_1 \wedge F_2$$
$$\forall v_1 \ldots v_k. F_1$$
$$\forall v_1 \ldots v_k. (F_1 \wedge F_2 \wedge ... \wedge F_n \Rightarrow F_{n+1})$$
$$\exists v_1 \ldots v_k. F_1$$

As discussed in Sect. 3, the structural sharing of goals means that the higher-level datatypes used by the program are different from those used in e.g. an LCF-style prover. The key datatypes are defined recursively as follows:

1. A *box* is either a *nontrivial box* or the special box $\top$.
2. A *nontrivial box* consists of a list of variables, a list of formulae (called *assumptions*) and a list of *targets*.
3. A *target* consists of either a formula or a list of boxes.

In the absence of variables, the box consisting of variables $v_1 \ldots v_k$, assumptions $H_1 \ldots H_n$ and targets $T_1 \ldots T_m$ corresponds to the aim of proving that the formula

$$\begin{aligned} \forall v_1 \ldots v_k. (H_1 \wedge \ldots \wedge H_n \Rightarrow T_1 \wedge \ldots \wedge T_m) \end{aligned}$$

holds. Where metavariables are present the corresponding quantifiers need to be existential rather than universal.

Targets that consist of a list of boxes correspond to a disjunction of the formulae corresponding to the individual boxes.

The goal consists of a single box, and tactics are functions that map boxes to boxes. In the rest of this document, we will display the box consisting of variables $v_1 \ldots v_k$, assumptions $H_1 \ldots H_n$ and targets $T_1 \ldots T_m$ as follows:Note that we suppress the variables as they tend to clutter the exposition; they are however present in the debug code produced by the program. Where a target consists of one or more boxes, we draw a rectangle around each box to delineate it.

We use the term *statement* to refer to a formula that appears either as an assumption or a target in some box. Statements and boxes may be tagged with additional information; for example, when a statement has been used together with certain other statements by a tactic, it is tagged to indicate this. Tags never affect the logical interpretation of the tagged material, but are used when the program is evaluating whether tactics are permissible. In particular, tags are used to prevent repeated application of the same tactic to the same statements.

Both the human-like output and debugging code prettify the output in conventional ways, for example by writing $a < b$ for the atomic formula $less\_than(a,b)$. We adopt such conventions throughout this document. In cases of ambiguity, quantifiers should always be read as taking the widest scope possible.

### Terminology

When a statement is built out of atomic statements using connectives and quantifiers, the program classifies it according to the operations that appear at the top of its parse tree. For example, the statement

$$\begin{aligned} \exists x\ (x\in A\ \wedge \ d(x,y)<\epsilon ) \end{aligned}$$

is an *existential* statement, whereas the statement

$$\begin{aligned} x\in A\ \wedge \ d(x,y)<\epsilon \end{aligned}$$

is *conjunctive*. It is often useful to look more than one level down the parse tree. For example, the program would call the statement

$$\begin{aligned} \forall x\ (x\in A\Rightarrow x\in B) \end{aligned}$$

a *universal conditional* statement. (Because we do not allow ‘bare’ conditional statements, this is a particularly important category). Similarly, the existential statement above can be further classified as an *existential conjunctive* statement.

Finally, many atomic statements can be expanded into statements that are no longer atomic. For example, the statement $A\subset B$ expands to the universal conditional statement above. It is often useful to know what a statement will become after it is expanded: to specify this the program uses the prefix ‘pre-’. Thus, the statement $A\subset B$ is pre-universal conditional. Similarly, the statement “*x* has an inverse” is pre-existential because it expands (in a suitable context) to

$$\begin{aligned} \exists y\ xy=yx=1 \end{aligned}$$

and the statement “*A* is unbounded” is pre-universal because it expands to

$$\begin{aligned} \forall C\ \exists a\in A\ a>C. \end{aligned}$$

An expansion is *elementary* if it does not introduce a quantifier. For example, the expansion of $A\subset B$ is not elementary, whereas the expansion of

$$\begin{aligned} x\in A\cap B \end{aligned}$$

as

$$\begin{aligned} x\in A\ \wedge \ x\in B \end{aligned}$$

is elementary.

### Substantive Hypotheses and Background Conditions

Consider the following definition. If *A* is a subset of a metric space *X* and $x\in X$, then *x* is an *accumulation point* of *A* if

$$\begin{aligned} \forall \epsilon >0\ \exists a\in A\ d(a,x)<\epsilon . \end{aligned}$$

An obvious way of rewriting this is

$$\begin{aligned} \forall \epsilon \ \ (\epsilon >0\implies (\exists a\ (a\in A\ \wedge \ d(a,x)<\epsilon ))). \end{aligned}$$

However, that does not reflect how a mathematician will think about the definition. The real number $\epsilon$ is not something that might conceivably be negative but happens to have a useful property if it is positive. Rather, when it comes to selecting $\epsilon$, the universe from which we select it is the set of positive real numbers. So the condition $\epsilon >0$ is not a ‘substantive’ assumption, but more like a background condition. By contrast, the statement $a\in A$ *is* substantive: it is an element of *X* that has the further interesting property of belonging to *A*.

We capture this in our program by representing background conditions through our type system. That is, instead of explicitly saying that $\epsilon >0$, we will take $\epsilon$ to have the type ‘positive real number’.

Note that this is important for the categorization of statements discussed in the previous subsection. For example, we want to think of a statement such as

$$\begin{aligned} \forall \epsilon >0\ \exists N\ \forall n\ge N\ d(a_n,x)<\epsilon \end{aligned}$$

as a universal existential statement and not a universal conditional statement. This is achieved by having the program represent it as

$$\begin{aligned} \forall \epsilon \ \exists N\ \forall n\ \ (n\ge N\implies d(a_n,x)<\epsilon ) \end{aligned}$$

and having the type system capture the fact that $\epsilon$ is a positive real number.

It is also important for deciding when a deduction is likely to be relevant. Suppose that we have a universal-conditional statement in the library that has several premises that match our assumptions. To mimic human reasoning, we would usually like this to count as evidence that the library statement is relevant, but not if the assumptions are merely background statements: if a library result requires a positive real number, we will not get excited just because we have a positive real number floating around.

### The Waterfall

The following lines are taken directly from the program’s code: they list, in order of priority, the names of the tactics it can use. (The names of the tactics do not always match the terminology used in this paper, which we have standardized to match the standard terminology used in interactive provers as far as we can). In Sects. 5.5–5.9, we shall describe each tactic in turn. 
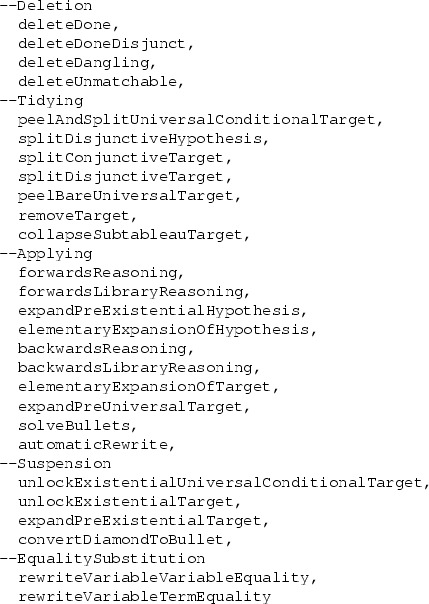


We stress here that because our system is fully automatic and intended to model human thought processes, our efforts have been concentrated less on the tactics themselves and more on how the program chooses which tactic to apply. For this program, the general form of the answer is as follows: it just chooses a tactic of the first type it can apply from the list above. Thus, if a tactic of type deleteDone can be performed, it performs it. If not, but a tactic of type deleteDoneDisjunct can be performed, then it performs that. Otherwise, it tries deleteDangling. And so on. In this way our architecture is similar to the ‘waterfall’ architecture used by Boyer and Moore in their provers NQTHM and ACL2. Like them we have tended to give higher priority to ‘lower-risk’ tactics, since this appears to correspond well to the way humans choose what to do; one of the challenges in generating human-like proofs is to assess correctly the risk of the different tactics. We discuss this point more fully in Sect. 5.10.

In the remainder of this section we shall describe the tactics, splitting them into some broad categories. One general preliminary remark is that a large proportion of the tactics contain minor ‘postprocessing’ steps, applying operations such as existential elimination. This is appropriate in precisely the cases where a human would apply such an operation in so automatic a fashion that it would not have any effect on the writeup. We will not mention these postprocessing steps in the tactic descriptions below as to do so would make them much longer. (Readers may of course consult the source code for full technical details).

### Removal Tactics

These tactics remove assumptions (cf. Sect. 3.3) and targets. They never directly contribute material to the writeup.

#### deleteDone

There are several situations where a tactic results in a target being replaced by $\top$ because it has been proved. Once this has happened, the program immediately uses this tactic to remove it. Thus, the aim of the program is to reach a goal with no targets.

#### deleteDoneDisjunct

If a target is disjunctive and one of its disjuncts is $\top$, then the entire target is removed.

#### deleteDangling

If an assumption has previously been used and contains a variable that is not involved in any other statement, then the program removes the assumption.

#### deleteUnmatchable

Roughly speaking, this tactic removes assumptions that have previously been used by a tactic and that have no obvious use.

For example, suppose that we have the statements $x\in A$ and $A\subset B$ as assumptions. The expansion of $A\subset B$ is $\forall u\ (u\in A\implies u\in B)$. If we substitute *x* for *u*, then the premise of this statement becomes $x\in A$, which is identical to the assumption. We say that $x\in A$ *matches* the premise of (the expansion of) $A\subset B$. We call a statement *unmatchable* if there are no available matches for it.

The program is not allowed to substitute the same variable twice into the same assumption. (This is partly because no human would ever do so, and partly to avoid non-termination). This can create further circumstances where an assumption is unmatchable. For example, suppose we apply forwards reasoning to the statements $x\in A$ and $A\subset B$ to deduce that $x\in B$. Then we can no longer use the match between $x\in A$ and $A\subset B$, so $x\in A$ becomes unmatchable (assuming that there is no other statement that matches it). Since it has been used, it will therefore be removed. If no other statement matches $A\subset B$, then that statement too is unmatchable and will therefore be removed, since it too has been used.

### Tidying Tactics

Tidying tactics are tactics that do not substantially change the content of a target, but put it into a more convenient form.

#### peelAndSplitUniversalConditionalTarget

If the target takes the form $\forall x\ (P(x)\Rightarrow Q(x))$, then this tactic removes it and adds instead a variable *x*, a new assumption *P*(*x*) and a new target *Q*(*x*). This corresponds to the human tactic of saying (or thinking), ‘Let *x* be such that *P*(*x*); we need to show that *Q*(*x*).’

The tactic could be thought of as a composition of two more basic tactics, one an application of universal generalization and the other an application of implication introduction, but again our avoidance of bare conditionals demands that we treat it as unitary.

If there is more than one target, then this tactic has to be modified, since we cannot use the new assumption *P*(*x*) to help us prove a different target. In that situation, we create a target that consists of a box.

That is, if we have a goal of the formthen the tactic will transform it toThe program can then use *P*(*x*) to prove *Q*(*x*) but not to prove *R*. The assumptions in the outermost box (which in the diagram above we do not enclose) can be used to prove both statements.

This tactic generates a representation which (if in isolation) would eventually be transformed into text like “Let *x* be such that *P*(*x*).”

#### splitDisjunctiveHypothesis

If there is an assumption of the form $P\vee Q$, then the program removes the disjunction by replacing the target(s) by two new targets. One of these targets is a box with *P* as an assumption and the original targets as its targets, and the other is a box with *Q* as an assumption and the original targets as its targets.

This tactic and its counterpart splitDisjunctiveTarget constitute work in progress; they are important for generating certain human-like proofs, but (because they split the proof into cases) they are not compatible with the incremental, concatenative tactic-by-tactic generation of writeups. We intend to extend the writeup generation mechanism to accommodate these tactics in future versions. Note that neither tactic is used in the problems we evaluate on.

#### splitConjunctiveTarget

If there is a target of the form $P\wedge Q$, then it is replaced by two targets *P* and *Q*. This tactic does not contribute to the writeup.

#### splitDisjunctiveTarget

If there is a target that consists of a formula of the form $P\vee Q$, then it is replaced by a target consisting of boxes corresponding to *P* and *Q*.

This tactic exists for technical reasons: one reason is that the program sometimes likes to attach tags to statements, for example to record whether they have been used (which affects the deletion rules), but it has no facility for attaching tags to parts of statements. Therefore, if we want to use a tag to record information about one disjunct of a disjunctive target, we need to ‘split’ the target first.

Also see the note regarding splitDisjunctiveHypothesis above.

#### peelBareUniversalTarget

If the target is of the form $\forall x\ P(x)$ and *P* is not a conditional statement, then this tactic removes the universal quantifier.

This tactic generates a representation which (if in isolation) would eventually be transformed into text like “Take $\epsilon > 0$.”

#### removeTarget

This tactic is formed from a family of related sub-tactics whose common element is that under appropriate circumstances they replace a target with $\top$. The most obvious example is when a target equals an assumption (and that assumption is allowed to be used to prove the target). A more complicated example is when the target is of the form $\exists u\ (P(u)\wedge Q(u))$ and there are assumptions *P*(*x*) and *Q*(*x*). The other circumstances are similar.

The sub-tactics generate representations that are eventually transformed into text like “Therefore, setting $\delta = \theta$, we are done.”

#### collapseSubtableauTarget

If a target consists of a single box *B* that has no assumptions and contains no metavariables (see Sect. 5.8 for a discussion of these), then the target is replaced by the targets of *B*. This tactic does not contribute to the writeup.

### Applying Tactics

An applying tactic is any tactic that corresponds to what human mathematicians would call applying an assumption, result or definition.

#### forwardsReasoning

The most basic form of this tactic is universal modus ponens: that is, a combination of substitution into a universal-conditional assumption followed by an implication elimination that uses the resulting conditional statement. In other words, one uses assumptions of the form $\forall u\, (P(u)\implies Q(u))$ and *P*(*x*) to obtain a new assumption *Q*(*x*).

A slight variant of this tactic that is worth highlighting is illustrated by the following simple piece of reasoning: if we know that $x\in A$ and that $A\subset B$ then we can deduce that $x\in B$. Humans will make this deduction in one step rather than first expanding the statement $A\subset B$ as $\forall u\ (u\in A\Rightarrow u\in B)$, then substituting *x* for *u*, and finally applying forward chaining. In order to produce a human-like writeup, our program does the same. In general, for each type of reasoning tactic that involves a universal conditional assumption, there is a variant that does the same thing to the expansion of a pre-universal conditional assumption.

This tactic also handles deductions that involve more than one premise, such as using assumptions of the form *P*(*x*), *Q*(*x*), and $\forall u\, (P(u)\wedge Q(u)\implies R(u))$ to obtain the assumption *R*(*x*).

This tactic generates a representation which (if in isolation) would eventually be transformed into text like “Since $x\in A$ and $A\subset B$, $x\in B$.”

#### forwardsLibraryReasoning

This is reasoning that is ‘mathematical’ rather than ‘purely logical’. For example, from the statements ‘$(a_n)$ is a sequence in *A*’, ‘*A* is closed’ and ‘$a_n\rightarrow a$’ one can deduce that $a\in A$. Experienced human mathematicians will perform this deduction in one step, because their mental library will contain the result that whenever a sequence in a closed set tends to a limit, then the limit belongs to the closed set as well. Mimicking this behaviour is very important for producing a write-up that is not cluttered with an inappropriate amount of detail.

Logically speaking, one could unify forwards library reasoning with ordinary forwards reasoning by adding the entire (allowable) content of the library to our list of assumptions. However, there are one or two aspects of library reasoning that give it a different flavour. The main one is that library results contain no free variables: they are general facts that apply universally. This distinguishes them from assumptions, which are more contingent. A second difference is that forwards library reasoning is normally used to deduce an atomic assumption from other atomic assumptions. A universal conditional statement is involved, but it is in the library and is not a assumption. For these reasons, reasoning that uses library results tends to be used by humans only when other forms of forwards reasoning are not available. Therefore, for the program it is important *not* to unify the two forms of reasoning, so that library reasoning can be given a lower priority.

It is also important to place some kind of restriction on forwards library reasoning to stop the program making obviously irrelevant deductions. For instance, if it knows that *H* is a subgroup of a group *G* and $x\in H$, and if a suitable result belongs to the library, then an unrestricted forwardsLibraryReasoning would allow it to deduce that $x^{-1}\in H$, but this deduction may well be of no help in solving the problem at hand and a step that no human would think of taking. As a safeguard against this, we forbid the program to apply the forwardsLibraryReasoning tactic if it creates a new term. This aspect of the program is not stable: it seems that although human mathematicians are indeed reluctant to create new terms in this way, they sometimes do so, even in some fairly straightforward problems. More work is needed to understand the circumstances under which such ‘speculative’ reasoning occurs.

This tactic generates a representation which (if in isolation) would be transformed into text like “Since $(a_n)$ is a sequence in *A*, *A* is closed and $a_n\rightarrow a$, $a\in A$.”

#### expandPreExistentialHypothesis

As its name suggests, this means replacing a pre-existential assumption by its definition expansion. (Recall that a statement is pre-existential if its definition expansion is an existential statement). What the name of the tactic does not reveal is that this expansion is followed immediately by an existential elimination. So for example expansion of the hypothesis ‘*A* is bounded’ might result in the introduction of a variable *M* and a new hypothesis $\forall x\ (x \in A \Rightarrow |x| \le M)$. We do this because human mathematicians almost always do it without comment, so our program should do so as well. Although these features have no effect on which problems the program can solve, they have a significant effect on the writing-up stage, saving the program from having to judge that certain steps do not need to be spelt out.

This tactic generates a representation which (if in isolation) would be transformed into text like “Since *A* is bounded, it follows that there exists *M* such that $|x| \le M$ whenever $x \in A$.” Note that, following standard human practice, in the rest of the write-up the variable *x* would automatically be treated as an entity one could refer to: the program is like a human in considering this kind of instance of ‘there exists *M* such that ...’ as equivalent to ‘let *M* be such that ...’. (Also note that a human typically suppresses the domain of quantification of *M* in this case, i.e. a human does not write $M \in \mathbb {R}$, and the program does the same.)

#### elementaryExpansionOfHypothesis

This takes a assumption that has an elementary expansion (recall that this means an expansion that does not begin with a quantifier) and replaces it by that expansion. This is sometimes combined with some tidying. For example, if the assumption in question is $x\in A\cap B$, then the elementary expansion is $x\in A\wedge x\in B$, but this expansion is immediately converted into the two assumptions $x\in A$ and $x\in B$ and does not itself appear in any intermediate goal—again so that the write-up will be suitably concise.

This tactic generates a representation which (if in isolation) would be transformed into text like “Since $x\in A\cap B$, $x\in A$ and $x\in B$.”

#### backwardsReasoning

This is the obvious backwards counterpart of forwards reasoning, using universal modus tollens instead of universal modus ponens. The most basic form is thus that we are given a target *Q*(*x*) and an assumption $\forall u\ (P(u)\Rightarrow Q(u))$, and we replace the target by *P*(*x*).

More generally, if we have a target *Q*(*x*) and an assumption $\forall u\ (P_1(u)\wedge \dots \wedge P_k(u)\implies Q(u))$, then it is logically sound to replace the target *Q*(*x*) by the *k* targets $P_1(x),\dots ,P_k(x)$, and many provers will happily do so. Our program is allowed to do this more complex backward chaining only under tightly constrained circumstances: we require all but one of the statements $P_1(x),\dots ,P_k(x)$ to be an assumption, so that only one new target is created. This is another pragmatic decision: it is a crude way of deciding whether applying the assumption $\forall u\ (P_1(u)\wedge \dots \wedge P_k(u)\implies Q(u))$ is likely to be the right thing to do, and the severity of the test is intended to stop the program making ‘speculative’ deductions that risk leading to combinatorial explosion. It is clear that humans sometimes violate this rule, but more work is needed in order to understand when they do so.

As with forwards reasoning, there is a simple variant where the role of the universal conditional assumption is played by a pre-universal conditional assumption instead. For example, given a target $x\in B$ and an assumption $A\subset B$ the program could use this variant to replace the target by $x\in A$.

The contribution of this move to the writeup is complex. An example of the output it can produce is “We know that $y\in A$ whenever $d(x,y)<\eta$.”

#### backwardsLibraryReasoning

This is backwards reasoning that makes use of a general result in the library. However, it is slightly subtler than forwards library reasoning, because it always uses assumptions as well as a target. The precise rule is that if there are assumptions $P_1(x),\dots ,P_{k-1}(x)$, a library result $\forall u\ (P_1(u)\wedge \dots \wedge P_k(u)\Rightarrow Q(u))$ and a target *Q*(*x*), then the target can be replaced by $P_k(x)$. (Of course, the premises of the library result do not have to be stated in the order $P_1,\dots ,P_k$).

An example of this kind of reasoning would be to say, “It is sufficient to prove that *B* is open,” if one wished to prove that $A\cap B$ was open and knew that *A* was open. This would be making use of the result that an intersection of two open sets is open.

Once again, the restriction we place is for pragmatic reasons: we do not want the program to make highly speculative transformations of the goal that introduce several new targets, since humans are usually reluctant to do this, especially when solving simple problems of the kind we are focusing on. But this is another situation where we would hope to improve the program in a future version, since humans do sometimes introduce multiple targets and then tick them off one by one.

The contribution of this move to the writeup is complex. An example of the output it can produce is “Since $d(x,y) < \delta$, $d(x,y) < \eta$ if $\delta \leqslant \eta$.”

#### elementaryExpansionOfTarget

This replaces a target by an elementary expansion of that target, if it has one. In the absence of metavariables, it generates a representation that will eventually be transformed into text like “we would like to show that *A* is open, i.e. that ...”. In the presence of metavariables, it generates a representation that will eventually be transformed into text like “we would like to show that $xy\in H\cap K$, i.e. that $xy\in H$ and $xy\in K$”.

#### expandPreUniversalTarget

This replaces a pre-universal target by its expansion. This tactic will be followed by one of the tidying tactics peelAndSplitUniversalConditionalTarget or peelBareUniversalTarget. It is usually the first tactic that the program applies when faced with a naturally stated problem.

This tactic does not generate any write-up text.

#### solveBullets

As we are just about to discuss in more detail, we sometimes convert a variable *w* into a metavariable. The metavariable needs at some stage to be chosen in such a way that the problem can be solved. If the variable only ever appears in targets, then one simple way in which this can often be done is to identify another variable *x* with the property that if we substitute *x* for *w*, then every target that involves *w* is equal to a assumption. In that situation, all those targets are replaced by $\top$.

This tactic generates a representation that will (after postprocessing) be transformed into text like “We may take $\epsilon = \ldots$ and we are done.”

#### automaticRewrite

There are a few term rewriting rules stored as data in the library. An example is that the term $(g\circ f)(x)$ is rewritten as *g*(*f*(*x*)). These rewriting rules are intended to represent operations that are so automatic that a human would not comment on them, and accordingly this tactic does not contribute to the writeup.

### Creation of Metavariables

We now come to a class of tactics alluded to earlier: tactics that help us deal with existential targets when it is not immediately clear what to substitute for the existentially quantified variable. A standard technique for this, which is essentially the technique we use, is to form *metavariables*. The rough idea of a metavariable is that one reasons with it as though it had been chosen, deferring the actual choice until later when it becomes clearer what choice will make the argument work. Mathematicians often use this trick: a classic example is the ‘$3\epsilon$-argument’ used to prove that a uniform limit of continuous functions is continuous.

We have found it convenient to introduce two kinds of metavariable, to model two styles of reasoning that are logically similar but psychologically quite different. As ever, this mimicking of human reasoning is necessary to produce a human-like writeup. These are displayed with diamonds or bullets, as described below.

#### unlockExistentialUniversalConditionalTarget

To illustrate this, suppose we have a target such as $\exists \delta \ \forall y\ (d(x,y)<\delta \Rightarrow f(y)\in B)$, and also a assumption $\forall u\ u\in A\implies f(u)\in B$. Then it is easy to see that we can reduce the target to $\exists \delta \ \forall y\ (d(x,y)<\delta \implies y\in A)$. However, this operation is not open to the program because it is not allowed to ‘reason inside quantifiers’. This is a matter of convenience: such operation are logically valid, but it is tedious to specify appropriate variants of several of the reasoning tactics listed above. Instead, we introduce a metavariable, which effectively moves aside the existential quantifier and allows the program to reason as normal with the statements inside it.

More precisely, what the program does to is replace the statement with a box whose variables include the metavariable that is being introduced. In the example above, it would have no assumptions and a single target $\forall y\ (d(x^\blacklozenge ,y)<\delta \Rightarrow f(y)\in B)$. The diamond on the (meta)variable *x* indicates that *x* needs to be chosen.

It is important for the program not to interchange quantifiers accidentally. For this reason, we tag the box just created with the variable $x^\blacklozenge$, to indicate the scope of the existential quantification over *x*.

After ‘unlocking’ the statement, the program applies the peelAndSplitUniversalConditionalTarget tactic inside the box. After that, we have a box that looks like this.Once we have done this, the statement $f(y)\in B$ has become an internal target and the program is free to apply backwards reasoning to it.

This tactic generates a representation that will (after postprocessing) be transformed into text like, “We would like to find *x* s.t. *P*(*x*) whenever *Q*(*x*).”

#### unlockExistentialTarget

This tactic replaces a target of the form $\exists x\ P(x)$ with a box that has the variable $x^\blacklozenge$, no assumptions and a single target $P(x^\blacklozenge )$.

This tactic will never be applied to an existential universal conditional target, since that will have been dealt with by unlockExistentialUniversalConditionalTarget. The main reason we have two separate tactics here is that we prefer to bundle the unlocking together with the peelAndSplitUniversalConditionalTarget tactic when that is possible.

To see what unlockExistentialTarget allows the program to do, suppose that we have a target of the form $\exists x\ (Q(x)\ \wedge \ R(x))$ and also a assumption of the form $\forall u\ (P(u)\Rightarrow Q(u))$. In this situation we would like to be able to do backwards reasoning inside the existential quantifier to reduce the target to $\exists x\ (P(x)\ \wedge \ R(x))$. However, the program does not have a tactic for this. Instead, it unlocks the existential target, so that it has a box with a target $Q(x^\blacklozenge )\ \wedge \ R(x^\blacklozenge )$. The tidying tactic splitConjunctiveTarget can now turn this new target into two targets, and once it has done that, the applying tactic backwardsReasoning can be used to replace the target $Q(x^\blacklozenge )$ by $P(x^\blacklozenge )$.

As another example of the use of unlocking, suppose that we wished to prove that $A\cap B$ is non-empty and had the assumptions $x\in A$ and $x\in B$. The program cannot see that *x* is a witness to the non-emptiness of $A\cap B$ without doing some processing. An obvious first step is to expand the target into the statement $\exists u\ u\in A\cap B$. However, the program is not then allowed to do an elementary expansion inside the quantifier. Instead, it unlocks *u* so that there is a new target $u^\blacklozenge \in A\cap B$. This can now be expanded and split into the two targets $u^\blacklozenge \in A$ and $u^\blacklozenge \in B$, which solveBullets can then match with the assumptions.

This may seem a little circuitous, but it actually models quite closely how humans think. A human might say, ‘I want to show that $A\cap B$ is non-empty, so I need to find some *u* that belongs to $A\cap B$. In other words, I need *u* to be in *A* and in *B*. Aha, I can take *x*.’ The program’s unlocking models the silent disappearance of the existential quantifier before the second sentence of the above, which we need to model to produce a human-like writeup.

This tactic generates a representation which will (after postprocessing) be transformed into text like “We would like to find *x* s.t. *P*(*x*).”

#### expandPreExistentialTarget

This does exactly what it says: it replaces a pre-existential target by its expansion. It generates a representation that will eventually be transformed into text like “We would like to show that ... .”, explicitly presenting the expansion.

#### convertDiamondToBullet

There are certain tactics that the program will not apply to a ‘diamonded’ metavariable. In particular, it will not do any reasoning with an assumption that involves such a metavariable: for that it needs another kind of metavariable, roughly speaking corresponding to the human operation of ‘pretending that a variable has been chosen’ and then reasoning with it. Logically this is not an important difference, but it is a useful one for us because it reflects a difference in the way human mathematicians think and write. This helps the program to produce more convincing write-ups. When we convert a ‘diamonded’ variable into a full metavariable in this way, we change the diamond to a bullet.

We do not need separate tactics for reasoning that involves assumptions with bulleted metavariables: we just allow the reasoning tactics described above to handle such metavariables.

An important technicality is that if we postpone the choice of a metavariable, we must keep track of which other variables it is allowed to depend on. However, what we actually do is note which variables it is *not* allowed to depend on. This is for two reasons. First, it seems to reflect more accurately how human mathematicians think about such variables, and secondly, it is more economical: there are typically many fewer variables on which a bulleted variable is not allowed to depend than variables on which it is allowed to depend.

This tactic generates a representation that will (after postprocessing) be transformed into text like “Assume now that ... .”, explicitly stating all assumptions involving the relevant metavariable.

### Equality Substitution

If we are told that two objects are equal, then we can eliminate all mention of one object in favour of the other. The precise rules governing when and how mathematicians tend to avail themselves of this opportunity are not obvious. The rules below are best regarded as a temporary solution: they do not always result in realistically human choices, and we intend to replace them by more satisfactory rules when we understand better what humans do.

#### rewriteVariableVariableEquality

If there is an assumption of the form $x=y$, then this tactic replaces all occurrences of *y* by *x* and eliminates the assumption.

This tactic generates a representation that will eventually be transformed into text like “Since $x = y$, ...”.

#### rewriteVariableTermEquality

If there is an assumption of the form $v=t$ or $t=v$, where *v* is a variable and *t* is a term, then this tactic replaces all occurrences of *t* by *v*.

This tactic generates a representation that will eventually be transformed into text like “Since $v = t, \ldots$”.

### Justification for the Order of Priority

As we have already said, the tactics we use above are all either standard in themselves or simple combinations of standard tactics (with the possible exception of our distinction between ‘diamonded’ variables and more standard metavariables). Our main concern is not the set of tactics available to the program, but the way the program chooses which tactic to apply to any given goal. We have attempted to design this so that the program can produce a realistically human style of output in an incremental fashion. That is, each tactic needs to produce a list of human-like English sentences, or more accurately a list of elements of a datatype that correspond to such sentences. The postprocessing described in Sect. 4 does not change the fact that the output of the program very closely matches its inner workings. This feature of the program has governed many of the design decisions we have made.

How should the program decide which tactic to use in any given situation? Our methodology for answering this question was to work through large numbers of problems ourselves, making a note of which tactics seem appropriate. After a while we were in a position to make a first guess at a suitable method for choosing tactics. We then tried the method out on more problems, adjusting it when it led to inappropriate choices. After several iterations of this, we arrived at the order of priority of the tactics that we set out in the previous section.

If our only justification for the order of priority were that it leads to good results for the problems we have tried so far, it would not be very strong: what gives us any confidence that the order of priority will be appropriate for other problems that may be quite different from the ones we have looked at so far? However, there is an informal guiding principle that explains quite a lot (though not all) of the order of priority, which is that the program prefers “safe” tactics to “dangerous” tactics. As we mentioned earlier, the same is true of the order of priority chosen by Boyer and Moore in their ‘waterfall’ architecture (see [12], p. 90).

Broadly speaking, a tactic is safe if the risk that it will lead to an undesirable result, such as a dead end or a step that is completely irrelevant to the eventual proof, is small. For example, tidying tactics are safe in this sense: by expressing the goal in a more convenient form, they open up new options without closing off any old ones. Since they are so safe, they come first in the order of priority. By contrast, expanding a definition is substantially less safe: sometimes it is possible to reason in a high-level way without expanding, and since we do not allow ‘de-expansion’ in this program (and in general allowing it would be highly problematic because of the danger of an infinite loop), expanding a definition is closing off the option of such high-level arguments, so we are reluctant to do it unless we have convinced ourselves that high-level arguments are not available. For example, if there is an assumption of the form ‘$(a_n)$ is Cauchy’, then we do not want our program to expand out the definition of Cauchy unless it has checked that it is not thereby closing off the option of a high-level deduction such aswhich would be a piece of forwards library reasoning in the program.

Thus, expansion has a fairly low priority. Having said that, some expansions, such as elementary expansions or expansions of pre-existential assumptions, are considerably safer, so those ones have higher priority.

Somewhere in between are the other reasoning tactics. Here it becomes more complicated to apply the general principle, even as an informal guide, because the safety of a tactic depends heavily on context. In particular, forwards reasoning is in general fairly unsafe—if you have a lot of information and do not know which statements are relevant, then the probability that any given deduction will form part of the eventual proof may be quite small— but it is much safer when it comes to routine problems, which tend not to suffer from the problem of irrelevant information.

It seems that *when it is safe*, humans tend to prefer forwards reasoning to backwards reasoning [27, 30], though this appears to be a question more of style than of problem-solving efficacy: we tend to prefer not to keep track of a moving target if we do not have to. Since forwards reasoning tends to be safe for the highly routine problems our program tackles, we have given all forwards reasoning a higher priority than all backwards reasoning. This also has the beneficial effect of making the program reluctant to switch direction—too much switching from forwards to backwards or vice versa would again be bad mathematical style.

This aspect of our program is, however, unstable, for the reason just given. When humans are faced with several possibilities for forwards reasoning, they will often switch to backwards reasoning in order to lessen the risk of making irrelevant deductions, but our program does not yet have any facility for making this kind of judgment.

One other feature of the ordering of reasoning tactics is that we prefer pure reasoning tactics to library reasoning tactics. That is because in general an assumption is more likely to be relevant than a library statement, though if enough of the premises of a library statement are present as assumptions, that is a fairly strong argument for its relevance.

At the bottom of the list of priorities is the process of creating metavariables. That is because humans tend to regard it as a last resort. When mathematicians need to prove statements of the form $\exists x\ P(x)$, then by and large they prefer to transform the goal using other tactics until a suitable candidate $x_0$ for *x* becomes obvious and it remains to carry out the relatively easy task of verifying that $P(x_0)$. Only when this straightforward approach fails do we take the more drastic step of pretending that *x* has been chosen.

We will not say much more here about how we chose the priority order, but we have two brief further points. First, although our reasons are not completely precise, we found that in practice they were adequate, in the sense that they suggested an order before we started, and we found that we did not have to modify the order when we tried further problems (though, as commented above, there are certain aspects of the architecture that will need to be changed in future versions). Secondly, when it comes to the finer detail of the ordering, there may not be that much to choose between different tactics. However, conflicts rarely arise between different tactics that are not distinguished by any of the above criteria, so in practice these finer details have little if any effect on what the program actually does.

## Example of Operation: An Intersection of Two Open Sets is Open

Now that we have discussed how the program works, let us look at another example, which involves most of the tactics we have discussed and shows how the order of priority works in practice. The problem to be solved is the following.

### Problem 1

Let *A* and *B* be open subsets of a metric space *X*. Prove that $A\cap B$ is open.

The initial goal is represented as follows.No reasoning tactics are possible, so we end up having to expand. The highest priority tactic we can do is expandPreUniversalTarget, which, after the tidying peelAndSplitUniversalConditionalTarget, has the following effect.Recall that we do not explicitly specify here that $\delta >0$, but instead take the positivity of $\delta$ to be part of its type. This is an example of why that is important: by suppressing background conditions such as $\delta >0$, we make it much easier for the program not to pay undue attention to them, and therefore easier for us to define our priority order in a unified way.

At this point, the program is trying to prove a statement that existentially quantifies over $\delta$. The nuclear option would be to convert the variable $\delta$ to a metavariable, but this operation has a low priority, so the program does as much forwards reasoning as it possibly can before resorting to it. It begins with elementaryExpansionOfHypothesis, applied to the third assumption.This allows it apply forwardsReasoning twice. After the first application, the goal is as follows.Note that the last assumption is in a sense generated by a combination of subtactics: the first is forwardsReasoning (using the assumptions $x\in A$ and ‘*A* is open’) and the second is an existential elimination (to get rid of $\exists \eta$ that would otherwise occur at the beginning of the statement). However, the latter is so automatic that it is not listed as one of our tidying tactics: instead, it is considered as part of any other tactic that potentially generates an existential assumption.

It is important to keep track of the fact that $\eta$ depends on *x*, which is what is signified by $\eta [x]$.

After this, deleteUnmatchable causes the program to remove the statements $x\in A$ and ‘*A* is open’. This is because both statements have been used and because it is no longer permissible to substitute *x* into ‘*A* is open’. The resulting goal is as follows.The program then runs through a similar process for *B* (it does not yet have the capacity to recognise that the problem is symmetric in *A* and *B* and say, ‘Similarly ...’). After that process, it arrives at the following.It has now reached the point where conversion of $\delta$ to a metavariable is the only option it has. In the first instance, it uses the tactic unlockExistentialUniversalConditionalTarget. The result is as follows.The notation $\delta ^\bullet [\overline{y}]$ signifies that $\delta$ is not allowed to depend on *y*.

The highest priority tactic the program can now apply is elementaryExpansionOfTarget, so it does that, and automatically splits the resulting conjunctive statement (rather than using the tactic splitConjunctiveTarget).This allows it to apply backwardsReasoning twice. After the two deductions it reaches the following state. (It does them separately, so we are jumping a step here).It then uses deleteUnmatchable to remove the two assumptions it has just used.At this point, there is not much that the program can do, because it is not allowed to reason with the diamonded variable $\delta ^\blacklozenge$. So the highest-priority tactic it can apply is convertDiamondToBullet. Also, since there are no assumptions above the main line, it replaces the goal by the inner box.Now it applies backwardsLibraryReasoning. The result in the library is that if $a<b$ and $b\le c$, then $a<c$. Applying that with the assumption and the first target results in the following goal.The removal tactics do *not* allow the program to remove the assumption we have just used (and this is a good example of a situation where deletion would be a very bad idea). However, it cannot use the assumption with the new target. The program then uses backwardsLibraryReasoning again and this time it does remove the assumption, on the grounds that the variable *x* that appears in the assumption does not appeat in any other statement. After that, it has reached the following state.This is a ‘standard’ existence problem, whose solution is stored as a construction in the library. The program uses this and declares the problem solved. It is here that the background information that $\delta$, $\eta$ and $\theta$ are positive is used, since the library result is that the minimum of two positive real numbers *a* and *b* is a positive real number that is less than or equal to both *a* and *b*.

## Testing the Write-Ups

Once the program had generated the write-ups for several problems, we wanted to test whether they could pass for write-ups written by a human mathematician. In this section we describe an informal experiment that we carried out for this purpose.

We began by asking two mathematicians, one an undergraduate and one a PhD student, to write out proofs for five problems for which our program had generated proof write-ups. We did not tell either of them why we were making this unusual request, and we did not ask them to make their write-ups as good as possible. One of the problems was to show that the inverse image of an open set under a continuous function is open, and one of our volunteers decided to prove the converse, so that he could use the topological definition of continuity to prove another of the assertions—that a composition of continuous functions is continuous. We had to ask him to rewrite the latter and give the epsilon-delta proof, since we wanted the differences between the write-ups to be a matter of style rather than substance.

We had another problem of this kind, which was that both our volunteers made frequent use of open balls. For example, their expansion of ‘$A\cap B$ is open’ was ‘for every $x\in A\cap B$ there exists $\delta >0$ such that $B_\delta (x)\subset A\cap B$.’ This made some of their arguments neater than the ones produced by our program. We contemplated getting the program to redo the problems using open-balls expansions, but in the end decided that it would be ‘cheating’ to make changes to its output in response to the human write-ups we had solicited, so we left things as they were.

The program’s write-ups were not designed to be indistinguishable from human write-ups: we merely wanted them to be acceptable as human write-ups. Therefore, we left in certain features, such as ending every proof with the words, ‘we are done’, that we could with a little trouble have changed. (See the brief discussion of non-determinism at the end of Sect. 4). For this reason, we did not want to ask people to guess which write-ups were by the program. Instead, we presented all fifteen write-ups—two by humans and one by the program for each of the five problems—on the second author’s blog, and asked readers of the blog to comment on them in any way they liked. We also asked them to award points for clarity and style. The orders of the write-ups were chosen randomly and independently. (The precise mechanism was to decide on a one-to-one correspondence between the set $\{1,2,3,4,5,6\}$ to the set of permutations of the set $\{1,2,3\}$, then to find a website that produced random dice rolls). So that answers would be as independent as possible, all comments and ratings were sent to the blog’s moderation queue and published only after the experiment was finished and comments on the blog post were closed.

The post can be found at http://gowers.wordpress.com/2013/03/25/an-experiment-concerning-mathematical-writing/, together with all the comments and ratings, but the real point of the experiment was to see whether anybody noticed that not all the write-ups were by humans. Nobody expressed the slightest suspicion of this kind.

Having said that, we should also remark that many commenters were highly critical of the program’s output. Three criticisms in particular stand out. First, as we expected, the fact that the program did not use open balls was unpopular: many people commented that this made the write-ups unwieldy. Secondly, several of the human write-ups stated the new target when the initial one had been stripped of universal quantifiers and conjunctions. Several readers commented that they found this helpful, and criticized our program for not doing it. And thirdly, commenters did not like the way the program spelt out in detail how it thought of the right variable to substitute into existential targets (such as choosing $\min \{\eta ,\theta \}$ for $\delta$ in the intersection-of-open-sets problem.

It would be easy to modify the program so that none of these criticisms apply, so they do not point to fundamentally non-human aspects of how it thinks. To change the first, we would just have to use a library containing open-balls expansions of definitions such as ‘*A* is open’ and ‘*f* is continuous’. To change the second, we could alter the rule for what the write-up does when we remove quantifiers and conjunctions, so that it states the new target (preceded by a phrase such as ‘We need to show that’). The third criticism would be harder to deal with, but in future versions, we plan to switch to having two styles of write up: a ‘proof write-up’ and a more detailed ‘proof-discovery account’. For the first style we will let the program work out the values of bulleted variables, then simply declare those values when the variable is first mentioned after being converted to a metavariable. This will correspond to the human practice of writing something like ‘Let $\delta =\min \{\eta ,\theta \}$’ or ‘Consider the sequence $(b_n)$ defined by $b_n=a_n/(1+a_n)$,’ which ‘magically’ does exactly what it needs to do later in the proof.

Although our program’s output came in for quite a bit of criticism, so did the write-ups by the undergraduate and PhD student—it seems that the readers were harsh judges. However, for most of the problems, the human write-ups were found preferable to the program’s.

After the success (as we considered it) of this experiment, we dared to try a direct test. We published a new post, this time explaining that one proof was by a program, one by an undergraduate and one by a PhD student, and inviting readers to vote on which one they thought was by the program. For each problem, the write-ups were numbered (a), (b) and (c). There were seven options for the voting: one could choose between (a), (b) and (c), but also choose between ‘The computer-generated output is definitely $*$’ and ‘I think the computer-generated output is $*$ but am not certain’; the seventh option was ‘I have no idea which write-up was computer generated.’ Again there was the opportunity to comment, for those who wanted to explain the reasons for their choices.

We did not reveal the results of the voting so far, or anybody’s comments, until the experiment was ended and the voting was closed. However, there was a different kind of dependence between answers, which was that people had the opportunity to look for clues that two different write-ups were from the same source. Given that we had not tried to remove stylistic ‘tics’ from our program’s write-ups, this put the program at a significant disadvantage. It was clear from the comments that many people had noticed that for each problem exactly one write-up ended with the words ‘we are done’.

Despite this, the program did reasonably well at fooling people that it was human. The typical pattern was that roughly half the voters would correctly guess which output was by the program, with slightly under half of that half saying that the output was definitely by the program. The undergraduate would always ‘come second’, and there would always be a fair number of people who said that they had no idea which output was written by the computer. There were surprisingly many votes for ‘The computer-generated output is definitely $*$,’ when $*$ was the wrong answer. The total number of votes was always at least 300, and for the first problem listed (the intersection of open sets is open) it was over 1000. One slight complication was that after a day or two the post was listed on the front page of Hacker News. The result was that the number of votes doubled in a couple of hours, and it may be that the profile of a typical voter changed. Fortunately, we had noted down the voting numbers just before this happened, so we presented those results as well as the final numbers. In the end, however, the proportions did not change very much. The detailed numbers can be found here: http://gowers.wordpress.com/2013/04/14/answers-results-of-polls-and-a-brief-description-of-the-program/.

One thing this experiment could not tell us, except to a limited extent through the comments, was whether the program was good at fooling *mathematicians* that it was human. It could be that the more mathematically experienced readers found the program’s output easy to distinguish, while the votes for the human write-ups came from people who were not used to reading mathematical proofs. However, we feel justified in concluding that the program’s output is not *obviously* written by a computer program, and that was our objective.

## Running the Program

The prover was written in Haskell, and contains about 3300 lines of source code. Readers who wish to replicate the evaluation or try the prover on other problems can obtain the source code at https://github.com/mg262/research/raw/master/robotone.zip; the readme file inside the archive contains instructions on compiling and running the prover. Note that although the problems and library are logically separated from the rest of the program, they are is currently stored as pure data in a Haskell module and compiled with the rest of the code.^3^ Output is produced as LaTeX source which is then compiled to two human-readable PDFs; one of these simply contains the proofs, and the other displays the step-by-step operation of the program with goals displayed at each stage.

Note that the shell script that invokes the prover also runs LaTeX on its output, and that this accounts for nearly all of its running time; the actual Haskell program runs too fast to measure (${<}$1ms) on the eight test problems included with the source code. This speed is a consequence of our aim of solving routine problems without backtracking or extensive search, just as a human does (Sect. 1.3).

Readers who wish to try the prover on other problems should be warned that the library *must* be tailored to the problem being solved.^4^ It is not possible to create a general, problem-independent library (without significantly modifying the program) because then the prover will use “more advanced”s results to prove simpler ones. For example, if one were simply to fill a library with every available result about real analysis and then ask the prover to show that $\sin$ is continuous, it could well deduce this from the fact that $\sin$ is differentiable and the fact that differentiable functions are continuous. But this is clearly an absurd proof.

This point may be illustrated with a problem tried by a referee, namely to show that a preimage of a closed set under a continuous function is closed. This problem was tried with the default library, which does not contain the requisite body of facts about sequences. In particular, the program contains the expansion
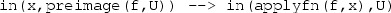


which allows ‘$x \in f^{-1}(U)$’ to be expanded into ‘$f(x) \in U$’. As with many other expansions, this rule has a direct analogue for sequences (and one for families, one for sets, etc.). Once that rule,
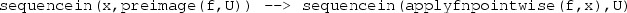


which allows ‘$(a_n) \in f^{-1}(U)$’ to be expanded into ‘$f((a_n)) \in U$’, has been added to the library, the prover produces a solution:

Let $(a_n)$ and *a* be such that $(a_n)$ is a sequence in $f^{-1}(U)$ and $a_n\rightarrow a$. Then $f(a_n)$ is a sequence in *U*. We would like to show that $a\in f^{-1}(U)$, i.e. that $f(a)\in U$ and since *U* is closed, $f(a)\in U$ if $f(a_n)\rightarrow f(a)$. Let $\epsilon > 0$. We would like to find *N* s.t. $\textit{d}(f(a),f(a_{n})) < \epsilon$ whenever $n\geqslant N$. Since *f* is continuous, there exists $\delta > 0$ such that $\textit{d}(f(a),f(a_{n})) < \epsilon$ whenever $\textit{d}(a,a_{n}) < \delta$. Since $a_n\rightarrow a$, there exists $N'$ such that $\textit{d}(a,a_{n}) < \delta$ whenever $n\geqslant N'$. Therefore, setting $N = N'$, we are done.

This solution is unsatisfactory in that it is operating at too low a level by reproving from scratch the fact that a continuous function preserves limits. Adding that result to the library gives a more satisfactory proof:

Let $(a_n)$ and *a* be such that $(a_n)$ is a sequence in $f^{-1}(U)$ and $a_n\rightarrow a$. Then $f(a_n)$ is a sequence in *U*. We would like to show that $a\in f^{-1}(U)$, i.e. that $f(a)\in U$. Since *f* is continuous and $a_n\rightarrow a$, we have that $f(a_n)\rightarrow f(a)$. Therefore, since *U* is closed and $f(a_n)$ is a sequence in *U*, we have that $f(a)\in U$ and we are done.

Note that if the library contains the the fact that a continuous function preserves limits, then the prover will generate a trivial proof when asked to prove that fact.

In cases where the program fails to solve a problem, the most likely cause is that the supplied library is not appropriate. Examining the final goal presented in the detailed output of the program usually makes it clear what fact(s) one has forgotten to include. Note that this is a benefit of our strategy of not backtracking: there is a definite single final state in which the program is ‘stuck’, and examining that state is invariably helpful.

## Future Work

In the short term, we would like to make a number of small improvements to the program so that it handles a greater range of problems satisfactorily. In the longer term, we would like to enlarge significantly the set of problems that our program, or some new version of it, is capable of solving. To do this, we will have to enable the program to handle certain kinds of deductions that it currently handles either not at all or only in a rather rudimentary way. In particular, an immediate target is to give the program the means to deal with second-order quantification, which would allow it to solve easy compactness problems, and also problems that require the construction of ‘obvious’ sequences.

At a more basic level, the program does not currently solve problems that involve proof by contraposition or contradiction. It is not hard to add tactics that allow it to cope with a few problems of this kind, but it is trickier to do so while not letting it apply those tactics in inappropriate contexts. More work is needed to understand what triggers the ‘contradiction move’ in human mathematicians, but we expect to be able to add this facility in the near future.

The program is also not as good as we would like at handling equality substitutions. The situation here is similar: we can obviously add tactics that perform such substitutions (and have done so in the current version of the program), but it is more challenging to understand when humans make such substitutions. It is also tricky to come up with a general understanding of how they choose which out of two equal variables or complex terms to eliminate. At its most general, the problem of how to handle equality is well known to be hard, but our immediate aim would be a program that can handle the easy cases of that problem competently and in a human way.

Related to this, we need to do more work in order to enable the program to solve problems that require the arithmetic structure of the real numbers, rather than just the order structure. For example, the prover does not yet solve problems such as showing that the limit of the sum of two convergent sequences is the sum of the limits.

In the longer term, we would of course like the program to be able to solve non-routine problems. A major step up in problem-solving sophistication is needed when one is required to carry out mathematical constructions, especially when they are far from unique. This is true even for very easy problems. Consider for example the problem of finding an infinite set of positive integers that contains no three distinct numbers *x*, *y* and *z* with $x+y=z$. One obvious example is to take the set of all odd numbers. Another that works for a different reason is to take the set of all powers of 2. Yet another, $\{1,2,4,7,12,20,\dots \}$ is obtained by taking each new element to be one more than the sum of the two largest elements so far. All these examples feel like ones that a human might conceivably come up with in response to the problem. We have ideas about how these kinds of simple (for humans) existence proofs are discovered, but implementing those ideas in a program will be a great deal of work.

## Footnotes

1 The program produces LaTeX output, which we reproduce verbatim here.

2 At a deeper level there are further differences. For example the Theorema system will perform a certain amount of backtracking even on routine problems such as that of showing that a limit of the sum of two sequences is the sum of the limits. Thus, while there are many points in common between our aims and those of the Theorema project, there are also important differences.

3 Using a Haskell module has allowed us to leverage Haskell’s excellent type-checking system to validate much of the input, and has also made it easy to construct specific libraries using higher-order operations during testing, with a considerable reduction in redundancy relative to a text format.

4 All of the data used by the prover, including the library used with the supplied problems, can be found in TestData.hs.

## References

[1] Asher N, Lascarides A (2003). Logics of Conversation.

[2] Ballantyne, A.M., Bledsoe, W.W.: Automatic proofs of theorems in analysis using non-standard techniques. J. ACM **24**(3), 353–374 (1977)

[3] Beeson, M.: Automatic generation of epsilon-delta proofs of continuity. In: Calmet, J., Plaza, J.A. (eds.) Proceedings of the International Conference on Artificial Intelligence and Symbolic Computation (AISC ’98), pp. 67–83. Springer, London, UK (1998)

[4] Beeson M (2001). Automatic derivation of the irrationality of e. J. Symbol. Comput..

[5] Bledsoe WW (1971). Splitting and reduction heuristics in automatic theorem proving. Artif. Intell..

[6] Bledsoe WW (1977). Non-resolution theorem proving. Artif. Intell..

[7] Bledsoe, W.W.: Set variables. In: Proceedings of the 5th International Joint Conference on Artificial Intelligence-Volume 1, pp. 501–510. Morgan Kaufmann Publishers Inc., San Francisco, CA. http://dl.acm.org/citation.cfm?id=1624548 (1977)

[8] Bledsoe, W.W.: Using examples to generate instantiations for set variables. In: Proceedings of IJCAI, vol. 83, pp. 892–901 (1983)

[9] Bledsoe WW (1995). A precondition prover for analogy. BioSystems.

[10] Bledsoe WW, Boyer RS, Henneman WH (1972). Computer proofs of limit theorems. Artif. Intell..

[11] Bledsoe, W.W., Hodges, R.: A survey of automated deduction. In: Shrobe, H. (ed.) Exploring Artificial Intelligence, pp. 483–541. Morgan Kaufmann Publishers Inc., San Mateo, CA (1988)

[12] Boyer RS, Moore JS (1979). A Computational Logic.

[13] Buchberger B, Crǎciun A, Jebelean T, Kovács L, Kutsia T, Nakagawa K, Piroi F, Popov N, Robu J, Rosenkranz M (2006). Theorema: towards computer-aided mathematical theory exploration. J. Appl. Log..

[14] Bundy A (2011). Automated theorem provers: a practical tool for the working mathematician?. Ann. Math. Artif. Intell..

[15] Clarke E, Zhao X (1992). Analytica—A theorem prover in Mathematica.

[16] Felty, A., Miller, D.: Proof explanation and revision. Technical Report MS-CIS-88-17, University of Pennsylvania (1987)

[17] Ganesalingam M (2013). The Language of Mathematics.

[18] Gonthier, G.: A computer-checked proof of the four colour theorem. http://research.microsoft.com/en-US/people/gonthier/4colproof.pdf

[19] Gonthier G, Asperti A, Avigad J, Bertot Y, Cohen C, Garillot F, Le Roux S, Mahboubi A, O’Connor R, Biha SO, Pasca I, Rideau L, Solovyev A, Tassi E, Théry L, Blazy S, Paulin-Mohring C, Pichardie D (2013). A machine-checked proof of the odd order theorem. Interactive Theorem Proving.

[20] Grice HP, Cole P, Morgan JL (1975). Logic and conversation. Syntax and Semantics.

[21] Hales, T., Adams, M., Bauer, G., Dang, D.T., Harrison, J., Hoang, T.L., Kaliszyk, C., Magron, V., McLaughlin, S., Nguyen, T.T., Nguyen, T.Q., Nipkow, T., Obua, S., Pleso, J., Rute, J., Solovyev, A., Ta, A.H.T., Tran, T.N., Trieu, D.T., Urban, J., Vu, K.K., Zumkeller, R.: A formal proof of the kepler conjecture. http://arxiv.org/abs/1501.02155 (2015)

[22] Holland-Minkley, A.M., Barzilay, R., Constable, R.L.: Verbalization of high-level formal proofs. In: Proceedings of Sixteenth National Conference on Artificial Intelligence, pp. 277–284 (1999)

[23] Humayoun, M., Raffalli, C.: MathNat—Mathematical text in a controlled natural language. J. Res. Comput. Sci.—Spec. Issue: Nat. Lang. Process. Appl. **46**, 293–307 (2010)

[24] Knott, A.: A data-driven methodology for motivating a set of coherence relations. Ph.D. thesis, University of Edinburgh (1996)

[25] Kuhlwein, D., Cramer, M., Koepke, P., Schröder, B.: The Naproche system. http://www.naproche.net/downloads/2009/emergingsystems.pdf (2009)

[26] Mancosu, P. (ed.): Mathematical explanation: why it matters. In: The Philosophy of Mathematical Practice, pp. 134–149. Oxford University Press, Oxford (2008)

[27] Owen E, Sweller J (1985). What do students learn while solving mathematics problems?. J. Educ. Psychol..

[28] Paulson LC (1987). Logic and Computation: Interactive Proof with Cambridge LCF.

[29] Reiter E, Dale R (2000). Building Natural Language Generation Systems.

[30] Sweller J, Mawer RF, Ward MR (1983). Development of expertise in mathematical problem solving. J. Exp. Psychol. Gen..

[31] Trybulec A (1978). The Mizar-QC/6000 logic information language. Bull. Assoc. Lit. Linguist. Comput..

[32] Vershinin K, Paskevich A (2000). Forthel—the language of formal theories. Int. J. Inf. Theor. Appl..

